# Acute lymphoblastic leukemia: an update on treatment and Brazilian perspective

**DOI:** 10.3389/fonc.2026.1637924

**Published:** 2026-02-13

**Authors:** Bruno Terra Correa, Gabriela Sales Serra Silva, Webert Joaquim Silva Mendes, Laryssa Lopes Soares, Joice Antunes Lima, Catarina Graziela Sousa Pereira, João Vitor Torquato de Souza, Maria Eduarda Freire dos Santos, Augusto Cezar Magalhães Aleluia, Caroline Conceição da Guarda, Regiana Quinto Souza Póvoas, Marilda de Souza Gonçalves, Milena Magalhães Aleluia

**Affiliations:** 1 Ciências Biológicas e da Saúde, Universidade Estadual de Santa Cruz, Ilhéus, Brazil; 2 Ciências Biológicas e da Saúde, Santa Casa de Misericórdia de Itabuna, Itabuna, Brazil; 3 Ciências Biológicas e da Saúde, Universidade Estadual do Sudoeste da Bahia, Vitória da Conquista, Brazil; 4 Laboratório de Investigação em Saúde Global e Doenças Negligenciadas, Instituto Gonçalo Moniz, Fundação Oswaldo Cruz, Salvador, Brazil

**Keywords:** acute lymphoblastic leukemia, cancer, oncohematology, pediatric, treatment

## Abstract

Acute lymphoblastic leukemia (ALL) is the most common pediatric cancer, representing a major global health concern, particularly in developing countries. This review provides an updated overview of the epidemiology, diagnosis, prognosis, treatment, and a Brazilian perspective for ALL. Despite advances in chemotherapy and immunotherapy, survival disparities remain challenging, driven by socioeconomic and healthcare access inequalities. Present diagnosis incorporates immunophenotyping, cytogenetics, and molecular biology for precise stratification and treatment planning. Treatment has evolved from monotherapy to multi-phase chemotherapy protocols, hematopoietic stem cell transplantation, monoclonal antibodies, and CAR T-cell therapy with risk-adapted strategies improving outcomes and offering hope in relapsed or refractory cases. However, these treatments remain largely inaccessible in low-to middle-income countries. Our review summarizes findings from Brazilian studies in children with ALL about diagnostics and treatment and highlights the importance of an accurate understanding of the national reality. Improved access to diagnostics, novel therapies, local multi-center studies, and pharmaceutical investment to enhance survival and quality of life for pediatric ALL patients is needed.

## 1 Introduction

Cancer is among the leading causes of death worldwide. In children and adolescents (0–19 years old), although the occurrence of cancer is rare, the most common type of cancer is acute leukemia, particularly acute lymphoblastic leukemia (ALL) (1).

Acute leukemias are aggressive, characterized by the cessation of differentiation and increased proliferation of hematopoietic progenitor cells (blasts), which accumulate pathologically in the bone marrow or, more rarely, in extramedullary sites (2). ALL, in turn, presents clonal pathological cells that accumulate in the bone marrow known as lymphoid progenitors (lymphoblasts) (2, 3). Due to the massive proliferation of lymphoblasts in the bone marrow, the differentiation and proliferation of normal hematopoietic cells cease to occur (4). Despite the new immunotherapies and the substantial improvement in chemotherapies since 1970, survival for ALL remains challenging.

The potential causal factors of ALL have not been fully established, although many chromosomal alterations and the generation of fusion proteins are likely involved in its pathogenesis (5). Embryonic genomics studies have found data indicating that 3 to 4% of children affected by ALL had a genetic predisposition to developing the disease. Alterations in germ lines associated with the *PAX5*, *IKZF1*, and *ETV6* genes are specifically associated with predisposition to the development of pediatric ALL (6). Constitutional syndromes, such as Down’s syndrome and ataxia-telangiectasia, are also associated with an increased risk of ALL since these children have a 20-fold increased risk of having ALL. The precise etiology of the increased risk in these syndromes has not been fully elucidated, however the literature suggests that mutations in the *CRLF2* and *JAK2* genes are involved in some cases (7).

Additionally, exposure to environmental factors is also a possible triggering factor for ALL. Still, few of them are potentially involved in an unequivocal way, such as ionizing radiation (8). Exposure to benzene (4, 9–11), to pesticides during pregnancy or after birth (12, 13), and viral infections (14–17) are also possible triggers.

The lifestyle habits of the parents may also be implicated in the occurrence of ALL, although there is no consensus in the literature. Alcohol abuse, illicit drugs, and smoking by the mother before conception and during pregnancy have been associated with an increased risk of developing ALL in the first 5 years of the child’s life (18). Nonetheless, some studies failed to identify a relationship between this consumption and the increased development of ALL (19). Thus, pediatric ALL is thought to have multifactorial causes, and environmental factors act as a trigger in those children who already have predisposing genetic alterations (20).

The disease’s symptoms are due to the lymphoblasts’ invasion of the bone marrow. Therefore, the patients develop anemia and symptoms such as pallor, tachycardia, dyspnea on exertion, and weakness, as well as leukopenia, which can lead to severe immunodepression and life-threatening infections. Additionally, patients exhibit thrombocytopenia, characterized by cutaneous and mucosal bleeding, including petechiae, gingival bleeding, and epistaxis (21, 22).

## 2 Epidemiology

The World Health Organization (WHO) funds and organizes a global study on the International Incidence of Childhood Cancer (IICC), and its latest version (IICC3) included five Brazilian studies (23). The data gathered in the IICC3 revealed that the incidence of pediatric cancer has been increasing over the years, with rates of 140.6 per million person/years in children aged 0 to 14 years, and 155.8 per million person/years in those aged 0 to 19 years. Moreover, ALL remains the most frequent cancer in the 0–19 age group, with a slightly higher incidence in males (23). In developing countries, the scarcity of data available on pediatric cancer and child mortality related to this disease makes it difficult to visualize its impact (1).

Regarding ethnicity, studies carried out in the USA show that the incidence of ALL is higher in Hispanics and lower in African descendants (23–26). Survival is usually lower in both groups when compared to American patients of Asian or European Caucasian descent (27). A multinational and multicenter genetic association study included patients from the USA, Latin America, and Southeast Asia and gathered crucial data regarding childhood ALL subtypes, ancestry, and mutations. There was a marked preponderance of T-cell ALL in children of African descent compared with those with a high percentage of Native American ancestry. In addition, African ancestry was associated with poorer event-free survival (EFS), overall survival (OS), and relapse (28).

The epidemiology, ancestry and survival of children with ALL still need additional investigation, although the Brazilian population bears one of the most significant genetic admixtures in the world. According to data from the National Cancer Institute (INCA), the estimated risk of childhood cancer for the period 2022–2025 is 135 per 1/1, 000, 000, 000. Despite this, the logistics of sending biological samples to these centers are still complex. In addition, a few national studies on pediatric ALL have been published in recent decades.

A prospective study with Brazilian children with ALL, who underwent induction therapy with the BFM 2009 protocol, has found that the contribution of Amerindian genomic ancestry was greater among children who developed severe general toxicity when compared to African and European ancestry for the occurrence of severe general toxicity (29). This data contrasts with the North American population, while in the USA, African children present a poorer outcome (27), in Brazil, Amerindian children may present more adverse events during the treatment. Larger cohorts are needed to corroborate these findings.

A previous cohort study was published following pediatric patients diagnosed with ALL-B between 2004 and 2009 in Bahia, treated according to the Brazilian Cooperative Group for Treatment of Childhood Acute Lymphoblastic Leukemia (GBTLI) protocol (version 93); a higher incidence was observed in males and an OS rate of 67.5%, 6 years after diagnosis. This survival rate is similar to what is found in other developing countries; however, it is lower than Europe and USA. They also point to the low number of specialized and trained professional reference centers in the state (30). This finding was corroborated by a current study, which states that excessive distance, coupled with the need to attend treatment frequently, results in worse survival outcomes, due to decreased adherence and increased drop-out rates (31).

A retrospective cohort study of patients treated at the blood bank reference center HemoRio, in Rio de Janeiro, published in 2022, revealed a 52% OS rate six years after diagnosis. This rate was partly due to the high number of adolescents, who usually present a more aggressive disease and are less responsive to chemotherapy treatment. In this group of patients studied, there was also a high number of patients with the presence of the BCR/ABL gene fusion, a genetic mutation associated with poor prognosis (32). Moreover, our group carried out a retrospective analysis involving 109 patients diagnosed with ALL and treated with the GBTLI protocol between 2010 and 2020. We found that after 5 years of diagnosis, the OS was 71.5%, and the EFS was 65% (33). Regarding age, a retrospective cross-sectional study was conducted in the Brazilian population between 2005 and 2015, and the authors identified 577 cases of ALL. From these cases, 325 (56.3%) were group aged ≤10 years, followed by 122 (21.1%) patients aged 11–20 years (32), which is consistent with other populations (34). These data reinforce the increased prevalence of ALL in younger children.

Immunophenotyping is essential for lineage classification (34). Findings from a Brazilian study have shown that 459 (79.55%) of the ALL patients were diagnosed with subtype B, 52 (9.01%) with T, and only one case was classified as biphenotypic (T/B) (35). Likewise, across five collaborating pediatric centers in four South American countries (Bolivia, Ecuador, Peru, and Paraguay), 92.1% of children under 18 years of age with ALL presented the B-cell subtype, while 7.5% had T-cell leukemia (36). A different study involving patients from Argentina, Bulgaria, Cuba, Chile, Croatia, Greece, Hungary, Poland, Serbia, Slovakia, Slovenia, and Uruguay has shown that 84.1% of childhood patients had B-type ALL, and 11.1% were classified as T-type ALL (37). Collectively, these findings suggest that, although slight differences may occur in the frequency of B or T-cell ALL, the frequencies observed in Brazil are comparable to those in other countries.

ALL remains a significant public health challenge in Brazil, primarily due to limited financial resources for diagnostic procedures in many regions and the scarcity of specialized reference centers across the country. As a nation of continental dimensions, Brazil is marked by profound socioeconomic and regional disparities, diverse ethnic backgrounds, and extensive miscegenation. Despite their methodological and regional heterogeneity, the available studies consistently report lower survival rates than those found in high-income countries. Larger, more comprehensive studies, with adequate epidemiological characterization, survival analysis, and identification of factors associated with relapse and mortality, are essential to guide more effective strategies aimed at improving outcomes in children with this highly curable disease, which can achieve cure rates exceeding 90% in developed settings.

## 3 Diagnosis

The 2016 WHO classification guidelines suggest that the ALL diagnosis must be defined based on cell morphology, immunophenotypes, genetics, and cytogenetics. Based on the suspected diagnosis, a blood count is taken, which shows anemia and cytopenias and may also indicate an increase in leukocytes (34). A bone marrow aspirate (myelogram) must be carried out to confirm the diagnosis. The aspirated material is stained, smeared on glass slides, and analyzed by microscopy to confirm the presence of lymphoblasts (38). The morphological characteristic of lymphoblasts consists of mononuclear, basophilic cells with very scarce cytoplasm. They can be similar to mature lymphocytes but are distinguished by their more immature chromatin and the occasional presence of nucleoli (39). According to the WHO, a diagnosis of ALL is confirmed when 20% or more of lymphoid blasts are found in the bone marrow (40).

Morphological diagnosis was initially based on the French-American-British (FAB) group classification, which considered cell size, cytoplasmatic and nucleolar characteristics, and the presence of basophilia. Three types of lymphoblasts were described: L1, L2, and L3 (41). Nevertheless, this classification had no predictive value. Later, in 1995, a classification method based on immunophenotyping was proposed (42). Since then, it has become possible to confirm whether the blast is indeed of lymphoid origin and whether the lymphoblasts are of B-lymphoid or T-lymphoid lineage. This test is carried out using the flow cytometry method, and material aspirated from the bone marrow or peripheral blood can be utilized when there are large numbers of circulating blasts (43).

Each hematopoietic cell line has specific antigens that characterize it, and, a nomenclature system, the Cluster of Differentiation (CD) system, has been created for these antigens. In the immunophenotyping method, pre-identified monoclonal antibodies labeled with fluorochromes can bind to the antigens expressed on the lymphoblast, thus, differentiating each cell type. The lymphoblasts of B-lineage precursor cells express CD10, CD19, CD22, and CD79a. Conversely, when there is an expression of CD3, CD7, and terminal deoxynucleotidyl-transferase (TdT), with the absence of expression of CD4, CD8, and myeloperoxidase (MPO), T-cell ALL is identified (4, 44). The cytogenetic study, performed at diagnosis to assess the karyotype of blasts, can reveal numerical chromosomal abnormalities, including the loss or gain of entire chromosomes, as well as structural abnormalities such as translocations, deletions, insertions, and inversions (4). The G-banding method is used for cytogenetic investigation, in which the blasts are exposed to mitosis inhibitors until they stop in metaphase. They are then subjected to hypotonic lysis with potassium chloride and fixed for later staining with Giemsa and digestion with trypsin (8, 43). Currently, there are molecular techniques such as FISH (Fluorescent *in situ* Hybridization), which allows for the precise localization of genes on chromosomes, and its variants, M-FISH (Multicolor FISH) and SKY (Spectral Karyotyping), which generate multicolored karyotypes and facilitate the identification of complex chromosomal rearrangements. There is evidence in the literature regarding a survey designed by the GBTLI LLA that 97.4% of services registered in the Brazilian Society of Pediatric Oncology (SOBOPE) for the treatment of pediatric ALL perform cytomorphological analysis of bone marrow as well as immunophenotyping (45). Indeed, in three different cohorts from Bahia, Rio de Janeiro, and Pará, immunophenotyping was performed in nearly 100% of the ALL children (29, 32, 33). The GBTLI survey also indicated that 92.2%perform cytogenetics and 87% have access to some diagnostic molecular biology [real-time quantitative polymerase chain reaction (RT-qPCR)] or fluorescence *in situ* hybridisation (FISH) (45). These data must be interpreted with caution, as these types of laboratory testing are not universally performed throughout the country and may be inaccurate for some centers in small cities (35). Moreover, from the 77 registered centers in SOBOPE that responded to the survey, only 23 (30%) perform the diagnostic examinations in an in-house setting (45). This is of particular importance due to the continental dimension of Brazil and its high heterogeneity among regions. In some centers, samples must be sent to a different city or state in order to run the cytogenetics, molecular biology, and FISH tests, which implies high costs and logistics challenges (30, 33).

Molecular biology studies are carried out using two methods: PCR and RT-PCR. The polymerase chain reaction is a tool for the specific amplification of nucleic acids (4). In this method, the genetic material is subjected to subsequent cycles of heat denaturation, polymerization, and consequent amplification of the segment of interest, using specific primers (8). In RT-PCR, gene fusion transcripts are processed through reverse transcription of messenger ribonucleic acid (mRNA), which enables the amplification and identification of equivalent DNA exons (46). Besides cytogenetics molecular biology studies are also carried out, which, can help identify certain alterations found in some leukemias. Their results can also influence the therapy of choice, risk stratification, and the search for MRD (8).

In 2008, the WHO defined a new, more comprehensive classification, which combined immunophenotyping findings with cytogenetic study results of lymphoblasts, describing alterations in the chromosomes of these blasts (47). In 2022, the fifth and most recent WHO classification for lymphoid tumors was published, which combines immunophenotyping, cytogenetic alterations of lymphoblasts, and genetic entities associated with new gene products that interfere with the cell’s functioning (48).

## 4 Prognostic and risk factors

Over the years, the increasing publication of studies involving pediatric ALL has made it possible to identify factors, at diagnosis, that indicate the likelihood of a favorable or unfavorable response to the proposed treatment. In terms of age at diagnosis, patients between the ages of 1 and 9 have a better prognosis (3,)and male patients tend to have worse results with treatment when compared to females (49). Regarding ethnicity, patients of Hispanic origin and African descent tend to have a more unfavorable outcome in North American populations (25).

The presence of lymphoblasts in the cerebrospinal fluid (CSF) at diagnosis is associated with a worse prognosis compared to the absence of leukemia invasion in the central nervous system (CNS). Thus, CNS involvement is a known risk factor for higher relapse and lower OS in children (50). The prevalence of CNS involvement at diagnosis in children with ALL in Brazilian analyses shows substantial variability. In two different studies carried out in Bahia, CNS involvement at diagnosis was 15.1% (30). Conversely, in a study developed in Rio de Janeiro, 12% of the patients presented CNS involvement (32), while a study with children from Belo Horizonte reported 1% (51). Differences in diagnostic approaches, regional epidemiology, disease biology, and patient demographics may explain the lack of consistency. In addition, CNS prophylaxis in association with systemic chemotherapy is highly recommended to avoid CNS relapse (34). Although this information is usually not clear in all the studies, a few of them describe the use of intrathecal chemotherapy (30, 32).

The characterization of lymphoblasts by immunophenotyping also has prognostic value, as T-lineage ALL usually presents a worse outcome, while B-lineage ALL presents a better outcome. Pro-B-type ALL usually has a worse prognosis due to its association with t(14;11). Contrarily, common ALL (CD10+) usually has a good prognosis (4). Another laboratory characteristic that implies a worse prognosis is the presence of hyperleukocytosis at the initial blood count. A leukocyte counts higher than 50, 000/mm3 in ALL-B and higher than 100, 000/mm3 in ALL-T indicates an unfavorable prognosis (34). The invasion of leukemic cells into the testicles can also indicate a poor prognosis (4).

Any alterations identified in cytogenetic and molecular biology studies can confer a favorable prognosis, as in cases of hyperdiploidy and the *ETV6/RUNX1* rearrangement, or can predict a poor prognosis, as observed in cases of hypodiploidy and the *BCR/ABL1* rearrangement (34). The DNA index is assessed by flow cytometry and estimates the number of chromosomes in the leukemia cell, a characteristic known as ploidy. It is defined as the ratio between the number of chromosomes in the leukemia cell and the number in normal diploid cells. When the ratio is higher than 1.16, it is associated with hyperdiploidy, which is generally associated with favorable prognosis (4, 52).

Additionally, minimal residual disease (MRD) has been recognized as one of the most important prognostic factors in previous decades. MRD refers to disease at levels undetectable by light microscopy (i.e., when no blasts are visualized in the bone marrow aspirate), but detectable by flow cytometry and molecular biology methods. The persistence of blasts (≥ 0.01% by flow cytometry or ≥ 0.001% by PCR) detected in the bone marrow at the end of the first month of treatment indicates a worse prognosis (53). MRD is an important prognostic factor for ALL, and different protocols guide the hematologists for testing it at several time points during the ALL clinical course. The MRD is assessed by flow cytometry or molecular biology techniques. A single-center experience in Southern Brazil, investigating children with B-cell ALL, has shown a significant association between the day 15 (D15) MRD detected through flow cytometry < 0.1% and the likelihood of dying or relapsing, and an association between D15 MRD > 10% and the likelihood of dying or relapsing. The cumulative hazard ratios for the relapse of patients with D15 MRD levels of < 0.1%, 0.1–10%, and >10% were 19.2%, 59.8%, and 80.1%, respectively (54). Additionally, a study conducted at the Hematology and Hemotherapy Foundation of Amazonas (HEMOAM) using flow cytometry to evaluate MRD in a cohort of 40 children with B-lineage ALL, the authors have shown that, upon diagnosis, 60% of the patients were identified as low-risk and 40% were high-risk patients (55). While the evaluation of MRD is reported in various regions of Brazil, its investigation is not widely available across all centers. Our group was able to perform MRD in only 15.6% of patients, as assessed at the end of the induction phase (33), and lack of MRD testing was also seen in different studies (32, 56).

## 5 Treatment

Once the diagnosis and prognosis of the disease have been adequately characterized, treatment should be started promptly. Before 1950, children affected by acute leukemia had a survival rate of weeks to a few months, and most of the time died due to infection or bleeding. Until then, there was no specific treatment, and the only therapeutic modalities available were blood transfusions and antibiotics (22).

In 1947, Dr. Sidney Farber, after noticing in his studies that folic acid supplementation caused a rapid increase in tumors in children with cancer, began therapeutic trials with folic acid antagonists for children with acute leukemia (22). Thus, he initially used aminopterin, an analog of methotrexate (MTX), in therapeutic trials with children with acute leukemia and obtained clinical improvement and hematological parameters in some patients with temporary remission of acute leukemia. This MTX analog is the first antimetabolite to demonstrate clinical antitumor activity (57). Based on these observations, specific chemotherapeutic treatments began to be used for acute leukemia.

In the following decades (the 1950s and 1960s), new chemotherapy drugs were developed, such as vincristine, daunomycin, and asparaginase, the latter currently considered essential in modern therapeutic protocols. Combination chemotherapy treatment protocols became the standard treatment for pediatric ALL (58). Between 1958 and 1962, studies were developed with the first treatment regimens for ALL with combined chemotherapy, showing remission rates ranging from 25% to 60%, at different treatment sites in the USA.

Initially, chemotherapy treatment protocols were divided into two phases: i) Remission induction, when patients were treated with vincristine, methotrexate, and cyclophosphamide. Later, prednisone was added in association with chemotherapy. ii) Maintenance, if the patient relapsed, a maintenance phase was added, with the usual use of chemotherapeutic agents 6-mercaptopurine and MTX. After these chemotherapy protocols, remission of the initial disease was achieved in around 40 to 60% of patients (59). Later, new phases were added to the so-called sequential chemotherapy protocols, including the “total therapy, “ leading to increased survival (60). The protocol introduced a new treatment modality for invasive lymphoblasts into the CNS, with intrathecal administration (injection into the CSF) of MTX. It is essential to note that the “*total therapy*” protocol represents a significant milestone in the history of pediatric ALL treatment, as it reduced CNS relapse and achieved a cure in approximately 50% of the treated patients (61).

In the 1970s, a cooperative study involving three cities in Germany (Berlin, Frankfurt, and Münster, BFM), gave rise to the BFM treatment protocol. The German group developed a sequential chemotherapy treatment protocol for pediatric ALL, including an earlier intensification phase and implementation of the reinduction phase, which led to an increase in the cure rate to around 70% (60, 62, 63). Importantly, the BFM protocol also established differences in the intensity and total treatment time, depending on the prognostic factors identified at diagnosis, the so-called risk stratification (63), providing a more personalized therapy. Patients identified at low risk of relapse through analysis of age, leukocyte counts, invasion of the CNS or testicles, and laboratory test results such as MRD research, cytogenetics, and molecular biology may be eligible to receive less intense chemotherapy and still have a high survival rate. Whereas, patients at high risk of relapse, must be treated with more intense chemotherapy, and face an increase in treatment-related toxicity, which can ultimately lead to greater lethality (34, 64). Therefore, the German treatment protocol remains useful in most pediatric oncohematology treatment centers worldwide, as risk stratification has been key to better outcomes.

Between the 1970s and 1980s, after noticing numerous side effects of radiotherapy on the craniospinal axis, studies were carried out to definitively replace radiotherapy with intrathecal administration of MTX, cytarabine, and hydrocortisone (later replaced by dexamethasone) (60). Equivalent results between craniospinal radiotherapy and intrathecal administration of chemotherapy and corticosteroids were found, which led to the gradual removal of radiotherapy from treatment protocols (65). The introduction of MTX chemotherapy, administered intravenously in intermediate doses, showed results comparable to those obtained with craniospinal radiotherapy for treating leukemia in the central nervous system, enabling effective treatment at this specific site without the unwanted effects of radiotherapy (66).

In 1980, the Brazilian Cooperative Group for the Treatment of Pediatric Acute Lymphocytic Leukemia (GBTLI) was created, which was responsible for implementing the first Brazilian treatment protocol for pediatric ALL, the GBTLI-80 (the first version was published in 1980, with a rationale similar to the protocols of the German BFM group). After the first results from the multicenter studies using the GBTLI-80 protocol were published, amendments were made, and new versions were created (GBTLI-82, GBTLI-85) with increased survival for children with ALL. In some Brazilian centers, OS reached 70% for children at low risk of relapse and 50% for children at high risk of relapse by the end of the 1980s (67). Thus, for relapsed patients (especially those with early relapse) or those with very poor prognostic factors, allogenic hematopoietic stem cell transplantation (HSCT) may be considered (4).

Allogeneic HSCT transplantation is an established strategy in the treatment of high-risk ALL in first remission, as indicated by the Brazilian Society of Bone Marrow Transplantation Guidelines, especially for patients with poor prognostic factors such as age over 35 years, t(9;22) positive, and persistent minimal residual disease after induction (68). In 1999, a study reported a series of 75 relapsed children with ALL, where 38 children underwent HSCT, and 37 were exclusively treated with chemotherapy. Disease-free survival was 62% in the transplanted group, compared to only 26% in those who received chemotherapy, after five years of follow-up. This data reinforces the superiority of the transplant in the scenario of relapsed children with ALL (69). While HSCT is a potentially curative treatment for ALL, particularly for high-risk patients and those with relapsed or refractory disease, its effectiveness is diminished by significant toxicity. Severe, potentially fatal complications can arise from HSCT. A retrospective, multicenter registry study included patients from 5 Brazilian reference centers, encompassing both public and private hospitals, aged ≥ 16 years with ALL or ambiguous lineage leukemia who underwent a first HSCT. The authors found an engraftment failure rate of 1.5%, a five-year cumulative incidence of chronic graft-versus-host disease (GVHD) of 26.2%, and a five-year overall survival of 40.7% (70). A different study has found a 19.5% of acute GVHD as a potential risk for early mortality in young adults (71). Conversely, a recent multicenter study analyzed outcomes of ALL patients aged up to 18 years who underwent HSCT. The authors found that transplants occurred in the first or second complete remission (CR) in 38.7% and 44.2% of cases, respectively. The 100-day cumulative incidence of acute GVHD was 46.1%, and chronic GvHD after 100 days occurred in 25.5% of the patients. At five years, the OS rate was 52.4%, while the event-free survival rate was 51.7% (72). ^469^These findings emphasize that HSCT harbors important adverse events and toxicity, which may be challenging to manage clinically, despite being one of the most important treatment approaches.

In the past decade, a study that followed children and adolescents with *BCR/ABL-positive* ALL showed that, in the long term, a tyrosine kinase inhibitor, imatinib, associated with chemotherapy was as effective as allogeneic hematopoietic stem cell transplantation. Therefore, it is suggested that children diagnosed with ALL and *BCR/ABL* positive no longer need to undergo transplantation as a first line of treatment (73). More recently, monoclonal antibodies, already present in oncohematological treatments such as multiple myeloma, Hodgkin’s and non-Hodgkin’s lymphomas, chronic lymphoid leukemia, and acute myeloid leukemia, have been considered a therapeutic option for ALL.

Immunotherapies and targeted therapy have been investigated for the treatment of ALL. Since ALL lymphoblasts may present CDs from either B-cell lineage or T-cell lineage, monoclonal antibodies are used, such as Daratumumab (anti-CD38), Rituximab (anti-CD20), Ofatumumab (anti-CD20), Blinatumomab (anti-CD19), Epratuzumab (anti-CD22), and Inotuzumab ozogamicin (anti-CD22) (Malard & Mohty, 2020). Monoclonal antibodies are indicated for relapsed-refractory disease. Blinatumomab, for instance, is a bispecific monoclonal antibody that binds in parallel to CD3 on T lymphocytes and CD19 on lymphoid leukemia cells. The binding to T lymphocytes increases cytotoxicity by binding to lymphoblasts, causing their lysis (74). Its clinical indication is usually reserved for relapsed or refractory disease to chemotherapy, with benefits even as a pre-transplant treatment for hematopoietic stem cells (75). Newly, the chimeric antigen T cell receptor (CAR T-Cell) treatment has played a substantial role in relapses (74). The anti-leukemic effect is exerted by laboratory-modified T lymphocytes expressing chimeric antigen receptors, which target CD19 present on lymphoid blasts, causing their lysis. A multicenter phase II study published in 2018 revealed an OS of 76% after 1 year of treatment in children and adolescents with relapsed or refractory ALL-B treated with tisagenlecleucel, an anti-CD19 CAR T-cell drug, proving the benefit of this modality in relapse or refractoriness (76). Currently, tisagenlecleucel was approved by the National Health Surveillance Agency (ANVISA, Brazil) in 2021 with an indication for pediatric and young adult patients up to 25 years of age with refractory B-cell ALL, or from the second relapse onwards. In late 2023, brexucabtagene autoleucel was approved by ANVISA for the treatment of patients who were non-responsive to prior therapies or those with recurring ALL. A longer follow-up of three years has shown that OS was 38.9 months for responder patients who received brexucabtagene autoleucel (77).

Improvements in chemotherapy protocols and a better understanding of treatment support have significantly contributed to an increase in the survival of pediatric patients with ALL through combined chemotherapy, CNS prophylaxis, risk stratification, HSCT, and immunotherapy. The OS rate in developed countries has reached approximately 90% after five years of diagnosis (78). In developing countries, however, these high survival rates are not usually observed, although chemotherapy treatment protocols are very similar to those used in developed countries (60). Studies in Argentina, India, and Brazil show OS rates of around 60 to 70% after five years of diagnosis, and the reasons for this inferior outcome are not fully established (60).

## 6 Brazilian perspective

Brazil is ranked 3^rd^ in the number of deaths by ALL. WHO is looking to add new therapies to the protocols and ways of reducing mortality rate. The Brazilian scenario for ALL presents challenges in diagnostics, treatment, and follow-up for patients. Many laboratory tests are not conducted at the patient’s treatment center. Delays between sample collection and receiving results can harm patients. Although cytomorphological analysis and immunophenotyping are performed in almost all patients, some specific analyses are outsourced to larger centers or hospitals, usually far from the small cities. The current reality in Brazil shows that L-asparaginase is an essential drug for treating the disease (79). An analysis conducted in Curitiba, Paraná, showed that patients treated with L-asparaginase formulations had survival rates exceeding 95%. The Oswaldo Cruz Foundation in Paraná and researchers at the University of São Paulo seek to develop new ways of using the enzyme to reduce adverse reactions and its effects. Differences of almost 10% in terms of OS were also seen in groups of children receiving different types of asparaginase. An investigation has shown that a group receiving type “A” of asparaginase had a three-year OS of 91.8%, while group “B” presented 83.8% (80). Until 2018, no form of L-asparaginase was registered in Brazil. The National Health Surveillance Agency (ANVISA) currently authorizes the drug. However, due to documentation of serious adverse reactions, Brazil has been facing difficulties obtaining the active ingredient, with no registered options. It is essential to avoid drug shortages in Brazil, mainly due to the high cost and difficulty of marketing, even though asparaginase is an active ingredient considered necessary by WHO (81). About the innovation therapy, this systematic review assessed PEG-asparaginase’s efficacy and safety in treating ALL in children and adolescents, comparing it with native L-asparaginase. Studies concluded that PEG-asparaginase could serve as a substitute for native L-asparaginase, showing a comparable profile (82). However, results found insufficient evidence to confirm significant advantages of PEG-asparaginase over native L-asparaginase in terms of efficacy and safety (82). It emphasized the need for new trials with rigorous methodologies to establish PEG-asparaginase as a superior drug in ALL pharmacotherapy for children and adolescents. In Brazil, PEG-asparaginase is used to treat ALL. However, its use is still limited in the SUS due to issues such as high cost, availability, and the need for formal incorporation into standardized protocols.

Indeed about the prophylactic cranial irradiation, studies suggest that augmenting total body irradiation with cranial radiation boost in patients with ALL with no prior CNS involvement did not improve relapse risk in central nervous system or survival outcomes (83). Prophylactic cranial irradiation has already been a frequent practice in the treatment of ALL, especially in children, as a way to prevent CNS infiltration. However, its use is currently highly restricted in Brazil and many other countries due to long-term side effects and advances in intrathecal therapies. It is a strategy used in cases of high risk of CNS relapse.

In Brazil, the centers usually treat patients with BFM or GBTLI protocols, and the drugs included in these protocols for chemotherapy are: 6-mercaptopurine, 6-thioguanine, cyclophosphamide, daunorubicin, dexamethasone, doxorubicin, etoposide, ifosfamide, L-asparaginase, methotrexate, prednisolone, prednisone, vincristine, and vindesine (32, 33, 84). The drugs are very similar to those used in different countries, such as India, Ireland, Tanzania, Thailand and Mali, except for some tyrosine kinase inhibitor and PEG-asparaginase, which are included in other protocols (45).

HSCT is still considered an alternative for patients at high risk of relapse. However, it is still a treatment with high complication rates (85). Treatment with CAR-T cell is an innovative therapy and is approved only for the treatment of patients up to 25 years old with refractory or relapsed B-cell ALL. Tisagenlecleucel was approved by ANVISA for the first sanitary registration in Brazil in February 2022, it has an estimated price of R$ 1.568.166, 09 (in Brazilian currency) (86). The clinical trial phases had the participation of Hospital Israelita Albert Einstein, with funding from SUS. The price of brexucabtagene autoleucel was not identified in public data. However, this therapy still has side effects such as cytokine release syndrome and neurotoxicity (85). Monoclonal antibodies and CAR T-cell therapies will also change treatment regimens, as can already be seen for relapsed or refractory disease. Nevertheless, the patient’s access to these innovative therapies remains challenging due to their high cost, difficulties in manufacturing and even difficulty in accessing them through a health maintenance organization. Although the two CAR-T therapies have received regulatory approval in Brazil, the SUS does not provide either of them (87). It means that if a patient with ALL is eligible for tisagenlecleucel or brexucabtagene autoleucel treatment, they are likely to attempt to access these therapies through a private healthcare system (health maintenance organization) or pay for them themselves. Private access still faces challenges in bureaucracy and a lack of standardization of the payment model. There is evidence in the literature that, as of 2024, fifteen children with B-precursor ALL have received tisagenlecleucel in seven different institutions in Brazil. After the infusion, five children relapsed, and six children remained disease-free after a median of 270 days of follow-up (88). ANVISA recognized and regulated the use of tisagenlecleucel in Brazil for pediatric patients and young adults up to 25 years of age in 2022, which made this type of application feasible in clinical practice. There is an ongoing national prospective registry study (NCT05541341) that includes approximately 200 Brazilian patients treated with tisagenlecleucel, with follow-up for up to 15 years (88). Blinatumumab was incorporated in the SUS in late 2022 for high-risk B-ALL children in first relapse, which means that, theoretically, all children with high-risk B-ALL may be eligible for the treatment (89).

In all treatments for ALL, a significant frequency of adverse effects is evident. However, chemotherapy and HSCT have higher relapse rates. As for CAR T-cell therapy, there are several obstacles in terms of side effects and the high cost of manufacturing (85), additionally, a few patients may relapse after the infusion It is essential to consider the search for quality of life, beyond survival, highlighting the essentiality of a holistic approach for patients with the disease. In addition, managing adverse effects needs to be under constant systematic surveillance and be the target of increasingly optimized treatment strategies (90).

The lack of uniform data from Brazilian centers for the study of ALL highlights the urgent need to coordinate oncohematology services for a multicenter publication about the epidemiologic patient profile of ALL in children. Publications driven by real-world data with standardized descriptions of ALL are needed.

Moreover, it is crucial to consider strategies that expand accessibility to these centers. Achieving the performance of diagnostic tests and the availability of established therapies is feasible, even in a country of continental dimensions like Brazil. Currently, the Advanced Therapy Nucleus (Nutera, from Portuguese *Núcleo de Terapia Avançada*) initiative is underway in Ribeirão Preto, São Paulo, aiming to develop a Brazilian CAR-T that SUS will provide at a lower cost than those offered by private companies. This type of strategy is imperative for middle and low-income countries to improve access to advanced therapies for their patients.

Collecting new data is essential to create a more accurate understanding of the national reality. Additionally, it is necessary to support institutions that investigate both old chemotherapy and novel advanced therapies. This will help improve treatment effectiveness, decrease adverse effects and relapses, reduce MRD, and enhance the quality of life for patients affected by ALL.
